# Triple-Layer Nanofiber Membranes for Treating High Salinity Brines Using Direct Contact Membrane Distillation

**DOI:** 10.3390/membranes9050060

**Published:** 2019-05-06

**Authors:** Mustafa Al-Furaiji, Jason T. Arena, Jian Ren, Nieck Benes, Arian Nijmeijer, Jeffrey R. McCutcheon

**Affiliations:** 1 Environment and Water Directorate, Ministry of Science and Technology, Baghdad 10001, Iraq; alfuraiji79@gmail.com; 2 Inorganic Membranes, Faculty of Science and Technology, Mesa+ Institute for Nanotechnology, University of Twente, P.O. Box 217, 7500 AE Enschede, The Netherlands; a.nijmeijer@utwente.nl; 3 Department of Chemical and Biomolecular Engineering, University of Connecticut, 191 Auditorium Rd. Unit 3222, Storrs, CT 06269-3222, USA; jta08003@uconn.edu (J.T.A.); jian.ren@uconn.edu (J.R.); 4 Films in Fluids, Faculty of Science and Technology, Mesa+ Institute for Nanotechnology, University of Twente, P.O. Box 217, 7500 AE Enschede, The Netherlands; n.e.benes@utwente.nl

**Keywords:** membrane distillation, triple layer composite membrane, highly concentrated solutions, PVDF, PES

## Abstract

A composite, three-layered membrane for membrane distillation was prepared from electrospun polyvinylidene fluoride (PVDF) nanofibers supported by commercial polyethersulfone (PES) nanofiber based nonwoven from E.I. duPont de Nemours company (DuPont). The membranes were tested in direct contact membrane distillation (DCMD) using a 5.0 M sodium chloride brine as a feed solution. The triple layer membrane combines the hydrophobicity of PVDF and the robustness of the PES. The triple layer membrane demonstrated excellent performance in DCMD (i.e., relatively high water flux compared to the commercial PVDF membrane and a complete salt rejection of the brine) with mechanical properties imparted by the PES layer. This work is the first to demonstrate the use of a commercially produced nanofiber nonwoven for membrane distillation.

## 1. Introduction

Membrane distillation is a thermally driven separation process in which only water vapor molecules transfer across microporous hydrophobic membranes. In direct contact membrane distillation (DCMD), a hot feed solution flows on one side of the hydrophobic membrane while a cold solution (normally DI water) flows on the other side generating the driving force (i.e., vapor pressure) across the membrane. DCMD has been considered as an alternative to traditional distillation to treat highly concentrated solutions and brines due to its low sensitivity towards salinity of the feed solution [1]. These brines may include reverse osmosis reject [2], produced water [3], forward osmosis draw solutions [4] and hypersaline lakes [5]. DCMD among other MD configurations has the advantages of simple design and ease of operation. However, the high heat loss by conduction across the membrane can reduce the performance of the process.

A suitable membrane for membrane distillation should have high porosity, low tortuosity, and high thermal, mechanical and chemical stability [6]. Conventional commercial membranes that are typically prepared by phase inversion have been investigated for the treatment of highly concentrated solutions by membrane distillation [1,7].

Electrospinning is a versatile technique that can be used to prepare nanofiber membranes at desired properties. Recently, nanofiber membranes have been considered for MD [8,9,10] because of their intrinsically high porosity and low tortuosity [11]. These properties facilitate vapor transport through a membrane yet often lead to a lack of mechanical strength which limit their usefulness to more conventional cast membranes [12]. 

Adding additional layers to a membrane, such as a supporting scrim, is common with membranes intended for pressurized membrane processes (such as reverse osmosis). Layering MD membranes for the purpose of increasing mechanical integrity has been less common. Prince and co-workers, for example, have studied multi-layered membranes for MD. One of their membranes consists of a top selective layer of electrospun PVDF nanofibers, a middle layer that is made of PVDF formed by immersion precipitation, and a bottom hydrophilic PET support layer [13]. Another three-layered membrane consists of a PVDF cast layer sandwiched between two PVDF nanofibers layers, one of which is modified to be hydrophilic [14]. Both membranes showed improved performance in MD over the nanofiber single layer PVDF membrane due to the increased liquid entry pressure of the triple-layer membrane. We borrow from these multi-layer approaches to examine the use of a commercially available nanofibrous nonwoven material from DuPont (DuPont PES) for membrane distillation. Using the DuPont PES, we apply PVDF nanofibers to both sides of the membrane and measure performance under relevant DCMD conditions. PVDF polymer was chosen because of its hydrophobicity and ease of preparation via electrospinning process. The resulting membrane is entirely nanofibrous, thereby retaining high porosity while garnering strength from the PES and hydrophobicity of the PVDF nanofibers on both sides.

## 2. Materials and Methods 

### 2.1. Materials

Sodium chloride (NaCl, crystalline, certified ACS) was obtained from Fisher Scientific (Pittsburgh, PA, USA). Acetone and dimethylformamide (DMF) were purchased from Sigma Aldrich (St. Louis, MO, USA). The polymer used in this study was Solef L3 polyvinylidene fluoride generously provided by Solvay Specialty Polymers (Alpharetta, GA, USA). PVDF-HVHP membrane with 0.45 μm pore size was purchased from Millipore and used as a control. This membrane has a porosity of 75% and a thickness of 105 µm. SEM image and other characteristics of the PVDF-HVHP membrane are given in [15]. Water used in this study was ultrapure Milli-Q (18.2 MΩ) water produced by a Millipore Integral 10 water system, (Millipore Corporation, Billerica, MA, USA).

### 2.2. Membrane Fabrication

To prepare the triple layer MD membrane, a solution of 11.1% PVDF dissolved in 4:1 DMF and acetone was electrospun directly onto a commercially produced nanofiber based nonwoven support from E.I. duPont de Nemours company (Wilmington, DE, USA). DMF alone is a good solvent for the PVDF. However, adding acetone to the solvent plays a key role in accelerating solvent evaporation due to its high vapor pressure leading to the formation of the electrospun nanofibers [16].

This nonwoven support was made of PES fibers spun using a proprietary technique. This material was selected as the support because it can be produced at scale, it has substantially higher strength than lab-made electrospun nanofibers, and the PES and PVDF have a common solvent in DMF. This last feature is important since the PVDF nanofibers will adhere to the PES support as residual solvent promotes fiber bonding. The finished structure consisted of a layer of PVDF fibers on both sides of the PES. 

The PVDF fibers were prepared using a lab-scale electrospinning system described in our previous studies [11,17]. The PVDF solution was dispensed through a 20 gauge blunt needle at 5 mL·h-1 with an 18kV potential and a tip-to-target distance of 26 cm. The needle oscillates side-to-side and the collector surface is a grounded rotating drum that was wrapped with the PES support. Spinning was done at room temperature and 65–75% relative humidity (controlled by compressed air). Three thicknesses were prepared: 85 microns, 115 microns, and 145 microns. The thickness of the membrane was controlled by the volume of the PVDF polymeric solution that was spun on both sides of the DuPont PES. As a control membrane, a mat consisting of only PVDF nanofibers with thickness of 85 microns was spun at 18 kV and 65–75% relative humidity.

### 2.3. DCMD Performance Tests

**Figure 1:**
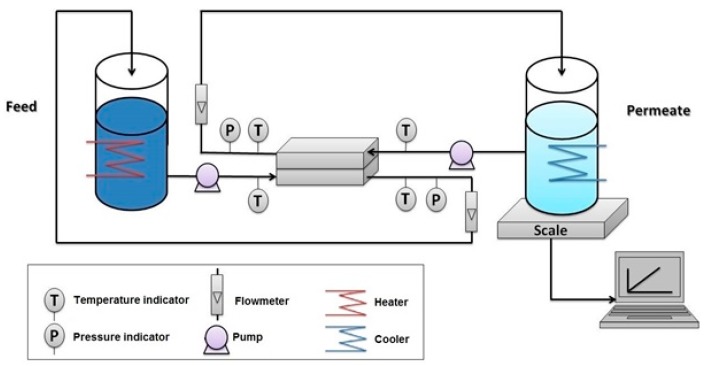
Schematic diagram of the direct contact membrane distillation (DCMD) bench-scale test unit.

The DCMD tests were carried out using a customized flat sheet membrane contactor system. The installation consists of two tanks: one for the feed solution and the other for permeate. A warm (50 °C) 5M NaCl solution was used as a feed in all experiment while DI water (20 °C) was used as permeate. Feed solution and permeate were pumped to the membrane cell using variable speed gear pumps (Cole-Parmer, Vernon Hills, IL, USA). A schematic diagram of the system is given in Figure 1. The flowrates of the feed and permeate were kept at 0.4 L/min. Four thermocouples, connected to a 4-channel temperature controller (Sper Scientific Direct, Scottsdale, AZ, USA) were used to measure the temperature at the inlets and the outlets of the membrane cell. 

The water flux was calculated based on the weight change of the collected permeate over the experiment time (6 h). The salt rejection R was calculated from the following equation:

(1) $$R=\left(1-\frac{C_{p}}{C_{f,i}}\right)\times 100\%$$

where C_f,i_ (g/L) is the initial concentration of the feed solution, and C_p_ (g/L) is the solute permeate concentration calculated from:

(2) $$C_{p}=\frac{C_{p,f}\left(V_{p,i}+\Delta V\right)-C_{p,i}V_{p,i}}{\Delta V}$$

In this equation C_p,i_, and C_p,f_ are the initial and final solute concentration at the permeate side, V_p,i_ is the initial permeate volume and ΔV is the permeate volume change during the experiment. The concentration of the solute in the permeate tank was calculated from measuring the conductivity of permeate using an YSI 3200 conductivity meter (Xylem Inc., Yellow Springs, OH, USA).

### 2.4. Membrane Characterization 

Scanning electron microscopy (SEM) was carried out using cold cathode field emission scanning electron microscope (FESEM, JSM-6335F, JEOL Ltd., Tokyo, Japan) to evaluate fiber size and the surface morphology of the PVDF and PES membranes. Prior to imaging, the samples were sputter-coated with a thin layer of gold. Imaging was done using an accelerating voltage of 20 kV and a current of 1.6 nA. Energy-dispersive X-ray spectroscopy (Thermo Noran System Six EDS, Thermo Electron Scientific, Madison, WI, USA) mapping was conducted using silicon drift detector series EDS (EDAX SDD-EDS). A CAM 101 series contact angle goniometer (KSV Company, Linthicum Heights, MD, USA) was used to measure the contact angles of water drops on the PVDF and the PES nanofibers at five different locations for each sample. The thickness of the different samples was measured at no less than five different locations of each sample using digital micrometer (Mitutoyo, series 293 IP65, Aurora, IL, USA). The mechanical properties of the different membranes were obtained from the tensile tests in air at 25 °C using an Instron microforce tester. A dynamic mechanical analysis (DMA) controlled force module was selected and a minimum of three strips (with a size of 40 mm × 5.5 mm) were tested from each type of membrane.

The porosity of the membranes was estimated using the gravimetric method. The membrane was cut into disks with a diameter of 2.54 cm (1 in) and weighed (W_dry_). Isopropyl alcohol (IPA) was used as a wetting agent and the membrane weighed after immersed in IPA (W_wet_). The porosity (ε) was calculated from the following equation [18]:

(3) $$\varepsilon =\frac{\left(\frac{W_{\mathrm{wet}}-W_{\mathrm{dry}}}{\rho_{\mathrm{IPA}}}\right)}{V}\times 100\%$$

where $\rho_{\mathrm{IPA}}$ is the density of IPA and V is the total volume of the sample. Each membrane was tested at least three times. The PVDF was assumed not to swell in the presence of IPA.

## 3. Results and Discussion

### 3.1. Membrane Characterization

Table 1 shows the properties of the membranes used in this research. The thickness was controlled by the amount of the PVDF polymer that was spun onto the PES layer. The data show that adding the PVDF nanofibers increases the average porosity from 60% to nearly 90%. The added layers of PVDF fibers to both sides of the PES layer increased the thickness of the membrane from 32 to 85, 115 and 145 microns). It can be also seen from Table 1 that the contact angle of the electrospun PVDF membrane is higher than that of the commercial PVDF cast membrane. This is attributed to the difference in the surface roughness as the electrospun nanofibers have ridge-and-valley structure leading to higher roughness compared to the PVDF-HVHP [6]. 

**Figure 2:**
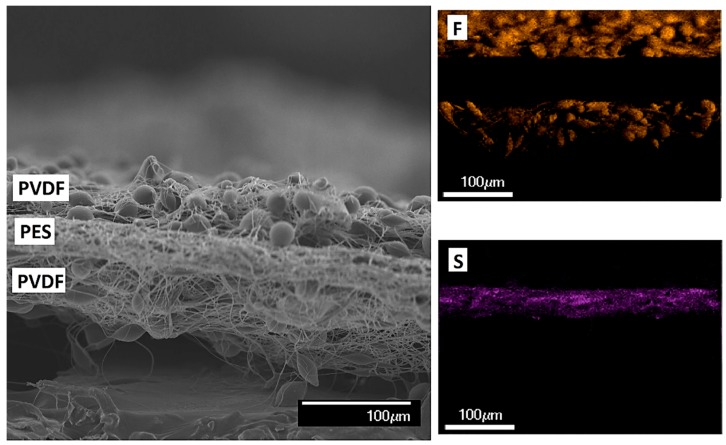
SEM image and energy-dispersive X-ray spectroscopy (EDS) mapping of the cross-section of the TL-115 membrane. Magnification ×230, the yellow color for the fluoride and the purple for the sulfur.

The cross-sectional SEM image and the EDS mapping of the triple layer membrane are shown in Figure 2. Here the three distinct layers of the triple-layered membrane can be seen, though it was difficult to section for the SEM imaging. We confirmed the presence of three distinct layers with a sharp interface using EDS mapping. The distribution of the fluoride and the sulfur through the cross-section of the membrane highlight the PVDF and the PES layers respectively. The cross-sectional image also shows the presence of beads in the fibers. Beads are generally not desired in many electrospun nonwovens since they can lead to a weakening of the fiber. In this case, the nanofibrous PES scrim negates this issue. The added roughness may also help prevent wetting of the membrane, but it would certainly be worth investigating further whether or not beads in electrospun fibers have a direct impact on MD performance with nanofiber membranes, which is beyond the scope of this study.

**Figure 3:**
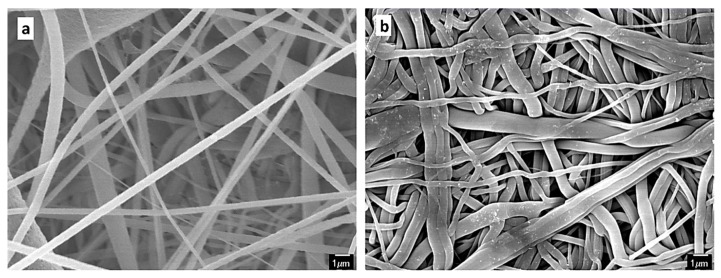
Surface SEM images of the (**a**) polyvinylidene fluoride (PVDF) and (**b**) polyethersulfone (PES) membranes at a magnification of 5000×.

The solvent in the PVDF solution (i.e., DMF/acetone) might result in dissolving or softening some of the nanofibers and enhances binding between the PVDF and PES fibers. The surface SEM images (Figure 3) shows the surface structure of the PVDF and the PES layers. It can be seen that the pore size of the PES layer is smaller than that of the PVDF layer. This is beneficial for MD as it increases the liquid entry pressure of water through the membrane [6]. The contact angle measurements in Table 1 confirm the hydrophobicity of the PVDF nanofibers and the relative hydrophilicity of the PES membrane.

**Figure 4:**
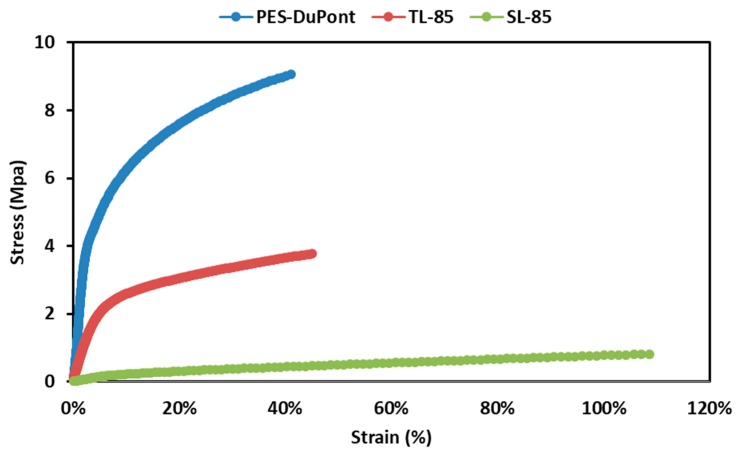
Stress-strain curve of the triple layer membrane, PES and the single layer PVDF membrane.

**Figure 5:**
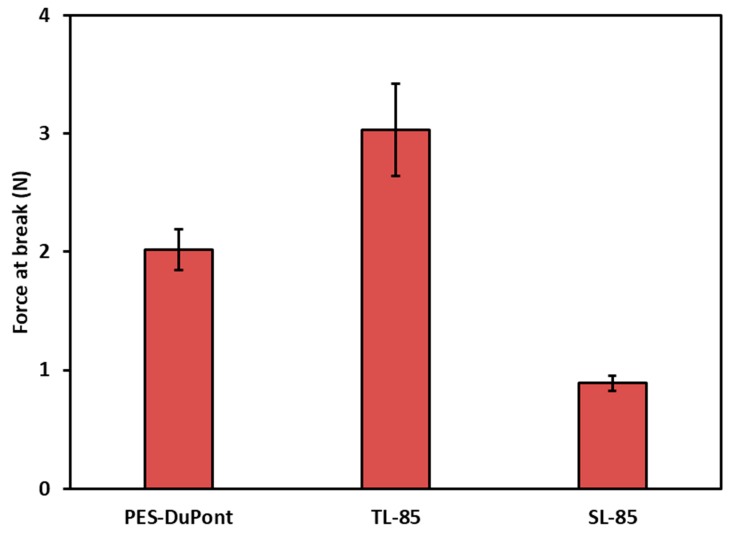
The force at break of the triple layer membrane, PES and the single layer PVDF membrane.

Mechanical properties of the membrane under tension are shown in Figure 4. The PES membrane alone has a tensile strength that far exceeds the PVDF nanofiber nonwovens on their own. Interestingly, after deposition of the PVDF nanofibers onto the PES, the membrane was weaker than the PES membrane alone. Essentially, the tensile test continues until reaching the tensile strength which is the maximum stress that can be achieved before breaking. This method measures the force at break divided by the cross-sectional area of the sample. The PES fibers have higher mechanical strength than the PVDF fibers; adding the PVDF fibers produces a material that has lower mechanical strength than the PES fibers alone. Normalizing for the cross-sectional area gives the force at break, which makes better comparison about the rigidity of the membrane. We present the same data but normalized for the cross-sectional area in Figure 5. Here, the triple layer membrane is shown to withstand a higher force at break and confirms its robustness over the PES or the PVDF nanofibers alone.

### 3.2. Membrane Performance by DCMD

**Figure 6:**
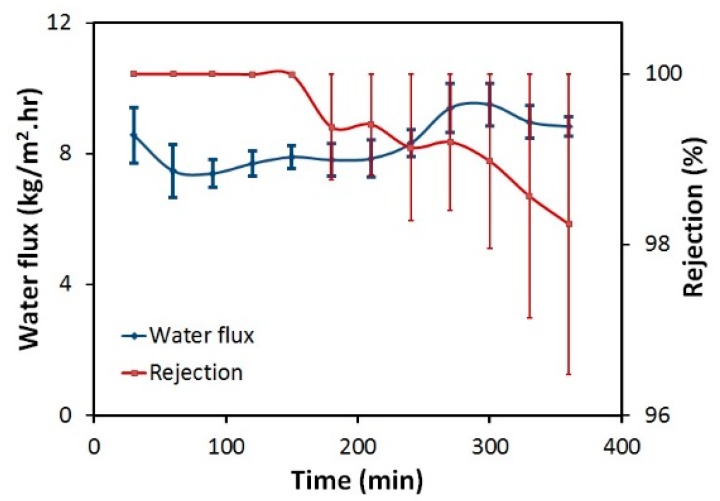
DCMD water flux and rejection for Dual-layer PES-PVDF membrane. Experimental conditions: feed solution: 5 M NaCl, permeate: DI water, feed temperature: 50 °C, permeate temperature: 20 °C, volumetric flow rate of feed and permeate 0.4 L/min.

DCMD performance was conducted for all the membranes using a 5 M NaCl feed at a 30 °C temperature differential. Testing on the bare PES membrane demonstrated no flux or salt rejection as the membrane wetted out almost immediately. We tested the PES membrane with a single layer of PVDF electrospun nanofibers (fibers on only one side) and the data is shown in Figure 6. While the rejection starts off high (approximately 100%, indicating a properly operating membrane), the rejection noticeably decreases after two hours. This demonstrates that wetting is occurring throughout the two-layer membrane. The large error bars in the rejection are indicative of the variability in the wetting, especially as wetting continues further. Given the relative hydrophilicity of the DuPont PES membrane, it is not surprising to see this variation. Interestingly, as salt rejection decreases, the water flux remains relatively constant. We attribute this to the fact that only small amounts of wetting are occurring as evidenced by the only small reductions in salt rejection. Such a small loss in salt rejection suggests that very little of the membrane is actually wetted from one side to the other. This small amount of salt flux across the membrane does not lead to a substantial loss of vapor pressure driving force, but the loss of rejection is easily detected by even the smallest wetted portion of the membrane since we are using a 5M NaCl feed solution. Similar behavior of dual-layer membranes was reported by Prince and his co-workers [13].

**Figure 7:**
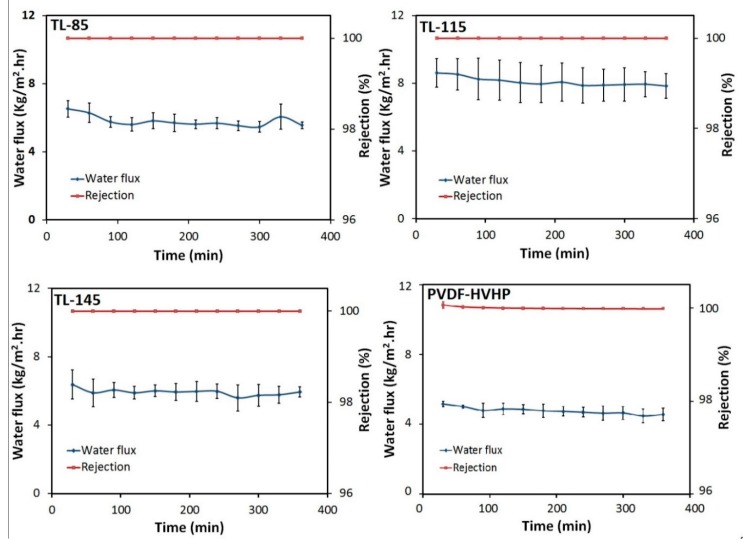
DCMD water flux and rejection for the prepared membranes with different thicknesses. Experimental conditions: feed solution: 5 M NaCl, permeate: DI water, feed temperature: 50 °C, permeate temperature: 20 °C, volumetric flow rate of feed and permeate 0.4 L/min.

The performance of all of the triple layer membranes is shown in Figure 7. The fluxes are between 6 and 9 kg/m^2^.hr and all membranes exhibit nearly complete salt rejection. The water flux and salt rejection were stable over 6 hours of operating time. In general, our triple-layer membranes exhibited a higher flux than the commercial PVDF-HVHP membrane. The TL-115 membrane showed a water flux twice as high as the water flux of the PVDF-HVHP membrane. This is due to the high porosity of the triple-layer membrane (around 90%) compared to the PVDF-HVHP membrane (75%). Porosity has been reported as the most influencing factor among other membrane properties [19]. Also, the triple-layer membrane showed comparable performance (water flux and salt rejection) compared to the results that were reported in the literature [7,13]. 

However, it is interesting to note that increasing the membrane thickness from 85 microns to 115 microns gives higher water flux but after increasing the thickness to 145 microns the flux dropped to about the same value. This can be explained by looking into the effect of the added thickness on the competition between heat and mass transport in membrane distillation. 

There is a trade-off between mass and heat transfer for the membrane with different thicknesses. A thinner membrane provides a higher rate of mass transport but it also increases the rate of heat transfer by conduction which increases the detrimental effect of temperature polarization [10]. Increasing a membrane’s thickness reduces the temperature polarization but also increases the mass transfer resistance. This is valid for a membrane that consists of a single material. The added layer in this research is highly porous. So, increasing the thickness of the membrane (from 32 microns to 85 or 115 microns) will increase the overall porosity and consequently the evaporation area and enhance the mass transfer. However, after adding more thickness (from 115 microns to 145 microns), the negative effect of the large thickness on mass transfer overcomes the positive effect of the high porosity and resulted in lower water flux. We note that in our system, we see the 115 micron thick membrane seems to balance the improved heat transfer resistance with a modest increase in mass transfer resistance and lead to the best water fluxes in this batch of membranes.

## 4. Conclusions

Triple layer nanofibers membranes with a commercial nanofiber mid-layer were investigated as a mechanically superior alternative to single layer nanofiber membranes. The membranes were comprised of a commercially available PES nanofiber nonwoven, having good mechanical strength, which was used to support a more hydrophobic PVDF electrospun nanofiber. The triple layer membrane showed better performance over both the PES and PVDF single layer membranes. The results also showed that the thickness of the membrane should be chosen carefully due to the interplay between increased heat and mass transfer resistance. When choosing materials for layered membranes, it is critical to understand this interplay and how it can affect performance. It is equally important to consider scalability of the fabrication process to ensure that such membranes have an opportunity to positively impact commercial desalination processes. 

## Figures and Tables

**Table 1:** Properties of membranes used in this research.

Membrane	Thickness (micron)	Porosity (%)	Contact Angle (°)
TL-85	85	87.8 ± 1	130 ± 2
TL-115	115	88.6 ± 4	128 ± 5
TL-145	145	88.8 ± 2	129 ± 3
SL-85	85	92 ± 2	132 ± 4
DuPont PES	32	60 ± 1.5	87 ± 1
PVDF-HVHP	105	75	115 ±7

TL: Triple-layer, SL: Single-layer.
